# Modeling health gains and cost savings for ten dietary salt reduction targets

**DOI:** 10.1186/s12937-016-0161-1

**Published:** 2016-04-26

**Authors:** Nick Wilson, Nhung Nghiem, Helen Eyles, Cliona Ni Mhurchu, Emma Shields, Linda J. Cobiac, Christine L. Cleghorn, Tony Blakely

**Affiliations:** 1 Department of Public Health (BODE3 Programme), Burden of Disease Epidemiology, Equity and Cost-Effectiveness Programme, University of Otago, PO Box 7343, Wellington, Wellington South New Zealand; 2 National Institute for Health Innovation and Department of Epidemiology and Biostatistics, University of Auckland, Auckland, New Zealand; 3 National Institute for Health Innovation, University of Auckland, Auckland, New Zealand; 4 University of Auckland, Auckland, New Zealand; 5 British Heart Foundation Centre on Population Approaches to NCD Prevention, Oxford University, Oxford, UK

**Keywords:** Sodium, Dietary salt, Targets, Economic analysis, Cardiovascular disease

## Abstract

**Background:**

Dietary salt reduction is included in the top five priority actions for non-communicable disease control internationally. We therefore aimed to identify health gain and cost impacts of achieving a national target for sodium reduction, along with component targets in different food groups.

**Methods:**

We used an established dietary sodium intervention model to study 10 interventions to achieve sodium reduction targets. The 2011 New Zealand (NZ) adult population (2.3 million aged 35+ years) was simulated over the remainder of their lifetime in a Markov model with a 3 % discount rate.

**Results:**

Achieving an overall 35 % reduction in dietary salt intake via implementation of mandatory maximum levels of sodium in packaged foods along with reduced sodium from fast foods/restaurant food and discretionary intake (the “full target”), was estimated to gain 235,000 QALYs over the lifetime of the cohort (95 % uncertainty interval [UI]: 176,000 to 298,000). For specific target components the range was from 122,000 QALYs gained (for the packaged foods target) down to the snack foods target (6100 QALYs; and representing a 34–48 % sodium reduction in such products).

All ten target interventions studied were cost-saving, with the greatest costs saved for the mandatory “full target” at NZ$1260 million (US$820 million). There were relatively greater health gains per adult for men and for Māori (indigenous population).

**Conclusions:**

This work provides modeling-level evidence that achieving dietary sodium reduction targets (including specific food category targets) could generate large health gains and cost savings for a national health sector. Demographic groups with the highest cardiovascular disease rates stand to gain most, assisting in reducing health inequalities between sex and ethnic groups.

**Electronic supplementary material:**

The online version of this article (doi:10.1186/s12937-016-0161-1) contains supplementary material, which is available to authorized users.

## Background

A diet high in sodium is ranked as the second most important dietary risk factor to health globally [1] and salt reduction is included in the top five priority actions for non-communicable disease (NCD) control internationally [2]. The World Health Organization (WHO) has a “strong recommendation” for countries to aim for a 30 % relative reduction in dietary intakes towards 5 g/day of salt [3].

While there are some persisting concerns around the relationship between dietary salt intake and health outcomes, the totality of the evidence is likely to justify public health action (as some of us have discussed elsewhere [4]). There are also several recent publications that add further weight to the adverse impact of dietary salt on health [5–10]. Even when the theoretical minimum level of risk exposure of sodium intake is modeled with wide uncertainty (from 1000 to 5000 mg of dietary sodium per day), it resulted in dietary sodium being ranked as the 11^th^ most important risk factor to health globally in the Global Burden of Disease 2013 Study [1].

There are now a number of modeling studies which have considered the health gain and/or economic aspects of dietary sodium reduction with reviews of these in the published literature [4, 11, 12]. Of these studies, some have started to consider reductions in sodium levels in specific targeted foods or food categories (Table 1). The results of these studies are all favorable in terms of generating health gain, and some report cost-savings. Nevertheless, there appears to be little modeling around evidence-based sodium reduction targets (i.e., that consider population sodium intakes and feasible reductions), or of comparing the relative benefits of achieving various food category specific targets e.g., for bread vs processed meats.

**Table 1:** Health impact and health economic modeling studies of population-level dietary salt reduction interventions involving reductions in sodium in processed foods (for publications from 1 January 2010 up to the end of June 2015 and ordered by publication year)

Setting and reference	Interventions aimed at specific foods/food categories	Main results/comment
Australia, Cobiac et al 2010 [47]	Voluntary and mandatory reduction of salt content in breads, margarine, and cereals.	Both were cost-saving interventions but health gain was much greater for the mandatory vs voluntary intervention (e.g., 110,000 vs 5300 disability-adjusted life-years [DALYs] averted).
US, Smith-Spangler et al 2010 [48]	Voluntary collaboration with industry was assumed to decrease sodium intake by the same amount as reported for the UK (9.5 %), with a range of 5 to 40 %.	Large health gain of 2.1 million QALYs, and savings in medical costs of $US32.1 billion (both over the cohort’s lifetime). Large benefits were seen with a salt tax.
Argentina, Rubinstein et al 2010 [49]	Voluntary reduction of salt content in bread by 1 g salt per 100 g.	Relatively small averted DALYs (compared to other CVD interventions) but still a cost-saving intervention. An earlier result by this team identified this intervention as cost-effective at ARS$151 (US$28) per DALY averted [50].
South Africa, Bertram et al 2012 [51]	Regulations to reduce the sodium content of bread, soup mix, seasoning and margarine (a reduction in salt of 0.85 g/person/day).	Substantial reduction in CVD deaths and non-fatal strokes estimated. Cost savings “of up to R300 million would also occur” (US$128 million).
Australia, Cobiac et al 2012 [52]	Mandatory reduction of salt content in breads, margarine, and cereals.	Large number of DALYs averted per year (80,000) and cost-saving. (See also a similar study listed above by these authors).
Argentina, Konfino et al 2013 [53]	Voluntary initiative currently in place in Argentina for 5 to 15 % reductions of sodium in: (i) processed meats, (ii) cheese and dairy products, (iii) soups and dressings and (iv) cereals, cookies, pizza and pasta.	Large reductions in: all-cause mortality, myocardial infarctions, and strokes, especially if the 2 year program agreed to with industry was extended to a larger 10 year one. (But no cost data included).
Netherlands, Hendriksen et al 2014 [54]	Theoretical reduction in salt in processed foods (variable by food category – but averaging 50 % reduction).	The median salt intake was expected to decrease by 28 % and blood pressure by 1.2 %. An estimated 256,000 DALYs were averted (239,700 to 272,300) and 0.15 per capita life years gained (0.11–0.19) among 40 year olds over the rest of their lifetimes.
New Zealand, Nghiem et al 2015 [4]	Mandatory 25 % reduction of salt in bread, processed meats and sauces. Also a voluntary endorsement label program (covering heart healthy foods [55]).	The gain was larger in the mandatory intervention (62,000 quality-adjusted life-years [QALYs]) vs the current voluntary endorsement label program (8000 QALYs). The interventions were pro-equity with relatively greater health gain for indigenous people (Māori).
USA, Choi et al 2015 [38]	Expansion of the National Salt Reduction Initiative to ensure all restaurants and manufacturers reach agreed upon sodium targets. These cut sodium in 62 categories of packaged, and 25 categories of restaurant, food items.	This expansion “would be expected to avert from 0.9 to 3.0 MIs [myocardial infarctions] (a 1.6–5.4 % reduction) and 0.5 to 2.8 strokes (a 1.1–6.2 % reduction) per 10,000 Americans per year over the next decade.” Most of the benefit came from changes in packaged foods. Also that “even high levels of consumer addition of table salt or substitution among food categories would be unlikely to neutralize this benefit”. The intervention was not found to reduce ethnic inequalities. No cost data were included.
England, Gillespie et al 2015 [56]	Mandatory reductions in all processed foods (10 % and 30 %). Voluntary reformation (24 %) – based on expert panel.	By the year 2025, maximum life-years gained by the mandatory reductions: at 43,900 for the 30 % and 14,800 for the 10 % levels. For voluntary: 14,300 life-years gained. The benefit in reducing health inequalities was greater for the mandatory than the voluntary interventions (when considering absolute differences in life-years).

Such information is important given that one of the approaches recommended by the WHO is food reformulation with “target setting” for food manufacturers [13]. Indeed, 13 European countries have “developed or are in the process of developing national targets for salt reduction, covering between one and 80 food categories” [14]. Moreover, some of these countries have already adopted mandatory targets (as maximal levels) e.g., Belgium, Bulgaria, Finland, Greece, Hungary, Portugal, and The Netherlands [14, 15]. There has also been progress around salt-reduction targets by nations in South America [16] and for South Africa [17]. Some of the best evidence for the benefit of reducing sodium levels in processed foods comes from the UK where sodium reduction targets, as part of a national sodium reduction strategy, have resulted in reduced sodium intakes at the population level [18–20].

Given this picture, we aimed to build on our previous modeling work [4] to estimate with uncertainty the health gain and cost-effectiveness of a country achieving the reduction recommended by WHO for dietary sodium intake, and also for the specific food category target components.

## Methods

### Model structure and perspective

The model was a Markov macro-simulation model in TreeAge Pro version 2013, from which we have previously published modeling interventions [4]. The simulated population was a closed cohort of the New Zealand population aged 35 years and older (2.3 million people), modeled from the baseline year (2011). The Markov model has four primary health states, with annual transition rates capturing incidence and case-fatality for coronary heart disease (CHD) and stroke events (see the diagram in an online Technical Report [21] on the BODE^3^ website; www.otago.ac.nz/bode3). The simulated population move through these health states until death or age 100 years, with the modeled intervention changing the annual transition probabilities of the population moving into these health states.

In terms of modeling background disease trends we took the same approach as the New Zealand Burden of Disease Study (NZBDS) [22], and assumed a continued decline in incidence rates for both CHD and stroke of 2.0 % annually, and also a 2.0 % reduction in case-fatality annually i.e., reflecting improved treatment and management. We extended this projection from 2016 (NZBDS end estimate) to the year 2026 and then held the incidence and case-fatality rates constant. Background population mortality was assumed to decline at a somewhat lower rate than for CVD with a 1.75 % annual reduction for non-Māori, and 2.25 % for the indigenous population of Māori (also out to the year 2026), then 0 % per annum decline for both ethnic groupings thereafter (for justification see: [23, 24]).

A health system perspective was used and costs and benefits beyond the health system (e.g., productivity gains from preventing premature deaths of workers) were considered out of scope. However, additional health system costs arising from extra life expectancy in the future attributable to the impact of the modeled interventions were included in the baseline analyses. Costs were calculated in 2011 New Zealand dollars and a 3 % discount rate was applied to costs and future health gain.

All interventions were evaluated against a theoretical “do nothing” comparator as per typical modeling practice [25]. This required us to remove the impact of the existing sodium reduction interventions in place in New Zealand of dietary counselling by dietitians and an endorsement food labeling program run by the Heart Foundation [4]. However, both of these interventions have relatively little impact on sodium intakes, and thus the analysis is fairly similar to a comparison with “current practice”.

### Input parameters

These parameters are summarized in the text below with additional details shown in Table A, Table B and Table C in Additional file 1.

### Incidence, prevalence and case-fatality

The estimated incidence, prevalence and case-fatality rates of CHD and stroke (ischemic and hemorrhagic) were calculated across all combinations of sex, 5 year age-groups (35–39, 40–44, … 95+ years) and ethnicity (Māori; and non-Māori). Data came from ‘Health Tracker’, which is a collection of linked administrative datasets of publically-funded health system events managed by the Ministry of Health [26]. Health Tracker includes hospitalizations, mortality, cancer registrations, mental health and addiction service use, pharmaceutical and laboratory claims, primary health care enrolment, and outpatient/emergency department visits for the entire New Zealand population with costs attached.

Validation of model parameters and the final model outputs (relative to two official data sources) are detailed in an online Validation Report [27]. This additional work also involved parameter coherence checking using the epidemiological software program DisMod II [28].

Subsequently, we also conducted a model validation exercise by comparing our TreeAge model with a multi-state life-table model, similar to the one used in a tobacco tax modeling study [29]. For the same sodium reduction intervention of a 22.8 mmol/day reduction in dietary intake (as used in our previous modeling [4]), the overall QALYs gained were 110,000 in our TreeAge model and 103,000 in the multi-state life-table model (both with 3 % discounting). We regarded this 6 % difference in results as acceptable given the models differed slightly in aspects of model structure and in baseline disease incidence rates and baseline case-fatality rates.

### Morbidity and disability weights

Overall morbidity, by sex, age and ethnicity, was quantified in the model using the years of life lived with disability (YLDs) from the NZBDS [22], divided by the population count to give ‘prevalent’ YLDs. Disease-specific morbidity was assigned in each disease state (e.g., CHD and stroke), as the total comorbidity-adjusted YLDs for that disease divided by the prevalent population. The health status valuation used to calculate these YLDs were disability weights (DW) derived from the Global Burden of Disease study (GBD2010) using pair-wise comparisons from multi-country surveys [30] (e.g., as opposed to using disutilities from the EuroQol). These DWs are on a scale from 0 (full health) to 1.0 (death) and include uncertainty (for details see the online Technical Report [21]). The quality-adjusted life-years (QALYs) were then cumulatively tallied for the life-span of the modeled cohort.

### Intervention specification

The “full target” and component food category targets were based on an evidence-based New Zealand salt reduction “target model”. Specific details of this work are in Table A in Additional file 1 and a thesis available online [31]. In brief, this “target model” involved estimating the sources of sodium in the typical New Zealand adult diet from a combination of market research company data on food purchases (electronically scanned by a consumer panel) and “Nutritrack” data for the brand-specific nutritional composition of packaged foods available in New Zealand supermarkets [32]. Salt consumed at home and in other foods away from the home was estimated from both National Nutrition Survey data [33], and US data [34]. Reductions in sodium in various food categories were partly informed by the UK Salt Reduction Targets for 2017, which had benefited from extensive consultation with the food industry [35]. The “full target” was a 35 % reduction in dietary salt (from 8.4 g to 5.5 g), to ensure an extra margin of success in meeting the WHO country recommendation of a 30 % relative reduction towards 5 g/day [3].

The interventions modeled are outlined in Table 2. For each of these we modeled either mandatory regulations requiring lower maximum levels of sodium in foods, or voluntary reductions. The latter had longer phase-in periods (5 vs 3 years) and more uncertainty around the impact (Table B, Table C in Additional file 1).

**Table 2:** Full target and component food category sodium reduction targets modeled

Intervention	Sodium reduction target details
1) Full target achieved (i.e., 35 % relative reduction to 5.5 g/d of salt) via packaged food target, fast food target and reduced discretionary use	The Intervention 2 target on all packaged foods (36 % reduction in sodium), plus the Intervention 3 target on fast food/restaurant meals (40 % reduction), plus discretionary use reduction (40 % reduction) but no changes to other foods. Overall there was a 35 % reduction.
2) Packaged foods target achieved	The specific targets for all packaged foods – including those packaged foods in this table and others in the full model [31] (a 36 % reduction in sodium in these foods overall).
3) Fast food and restaurant target achieved	An overall reduction of 40 % in sodium in these foods.
4) Bread target achieved^a^	Targets ranged from a 12 % reduction in wholemeal bread to a 37 % reduction in “other bread”. The most common target was to reduce to 350 mg sodium per 100 g of bread. A systematic review has indicated that salt can be reduced by approximately 40 % in breads [57].
5) Processed meats target achieved^a^	Targets ranged from a 35 % reduction in “cured meats” to a 55 % reduction in “other meat products” (covering all categories except for “raw” and “frozen” meat). A systematic review has indicated that salt can be reduced by approximately 70 % in processed meats [57].
6) Sauces target achieved^a^	Targets ranged from a 30 % reduction in marinades to a 63 % reduction in “powdered mixes for meal-based sauces”.
7) Package of Interventions 4 to 6	The combined (fully additive) effect of achieving the targets for bread, processed meats, and sauces collectively (the top three contributors to dietary sodium).
8) Snack food target achieved^a^	Targets ranged from a 34 % reduction in “extruded snacks” to a 48 % reduction in “potato chips”.
9) All bread and bakery target achieved^a^	As per Intervention 4 but with all other bakery products added (54 % reduction in “sweet biscuits” and 63 % reduction in “cakes, muffins and pastries”).
10) Cheese target achieved^a^	Targets ranged from a 27 % reduction in “hard block cheese” to a 42 % reduction in “soft/fresh cheese”.

^a^ This specific intervention was a component of Intervention 2, which in turn contributed to the “full target” in Intervention 1

### Costing of intervention scenarios and health system costs

We considered the net cost, which is the intervention costs plus health system costs throughout the lifespan of the modeled cohort (i.e., the results captured additional health costs associated with any extra lifespan generated by the interventions). Specific details for the costing of the interventions are provided in Table C in Additional file 1. For health system costs, the ‘business-as-usual’ ones were determined by strata of sex and age using Health Tracker data, which links cost estimates to all health events. From this dataset we calculated the 2011 costs for the first year of CHD and stroke, and then the average annual cost for the second and subsequent years. Furthermore, given that CVD is a relatively important part of baseline health system costs, we adjusted the baseline health system costs experienced by the “healthy” component of the modeled population, to remove the CVD-attributable cost component (to avoid double-counting).

Of note is that gaps in Health Tracker data exist in specific areas (e.g., some private sector expenditure and the health-related aspects of residential care [26]) and so we scaled up both the CVD disease costs and the annual health system costs for the non-diseased population. For the disease costs we scaled up Health Tracker costs across all age groups by 1.2, given that 83 % of all health spending in New Zealand is public (i.e., 1/0.83 = 1.2). Finally, costs at older ages were multiplied by 1.1, 1.2, 1.3 for the 65–74, 75–84 and 85+ age groups respectively to capture the estimated missing data of residential ‘disability support services’ care funded through government (‘Vote:Health’) but not yet captured in available data. All costs included those in the last 6 months of life.

### Analysis

For each of the interventions a reduction in dietary sodium intake was linked to a reduction in systolic blood pressure (BP) based on values derived from the regression models developed by Law et al [36] (see Table A in Additional file 1 for details). A reduction in systolic BP was then linked to a reduced probability of adverse health outcomes (CHD and stroke) as per a meta-analysis of 61 prospective studies by Lewington et al [37]. From this we generated QALYs gained and changes in health costs (from reduced disease burden and increased life expectancy). Analyses were by sex, ethnicity, and age-group.

We reran models (usually for expected values only) for a range of scenarios to assess the impact of components of the interventions and other structural assumptions (e.g., the discount rate: at 0 and 6 %). As an additional scenario analysis for the voluntary interventions, we modeled an effect size that was half of the baseline values to account for plausible non-compliance with the food category targets.

## Results

**Fig. 1:**
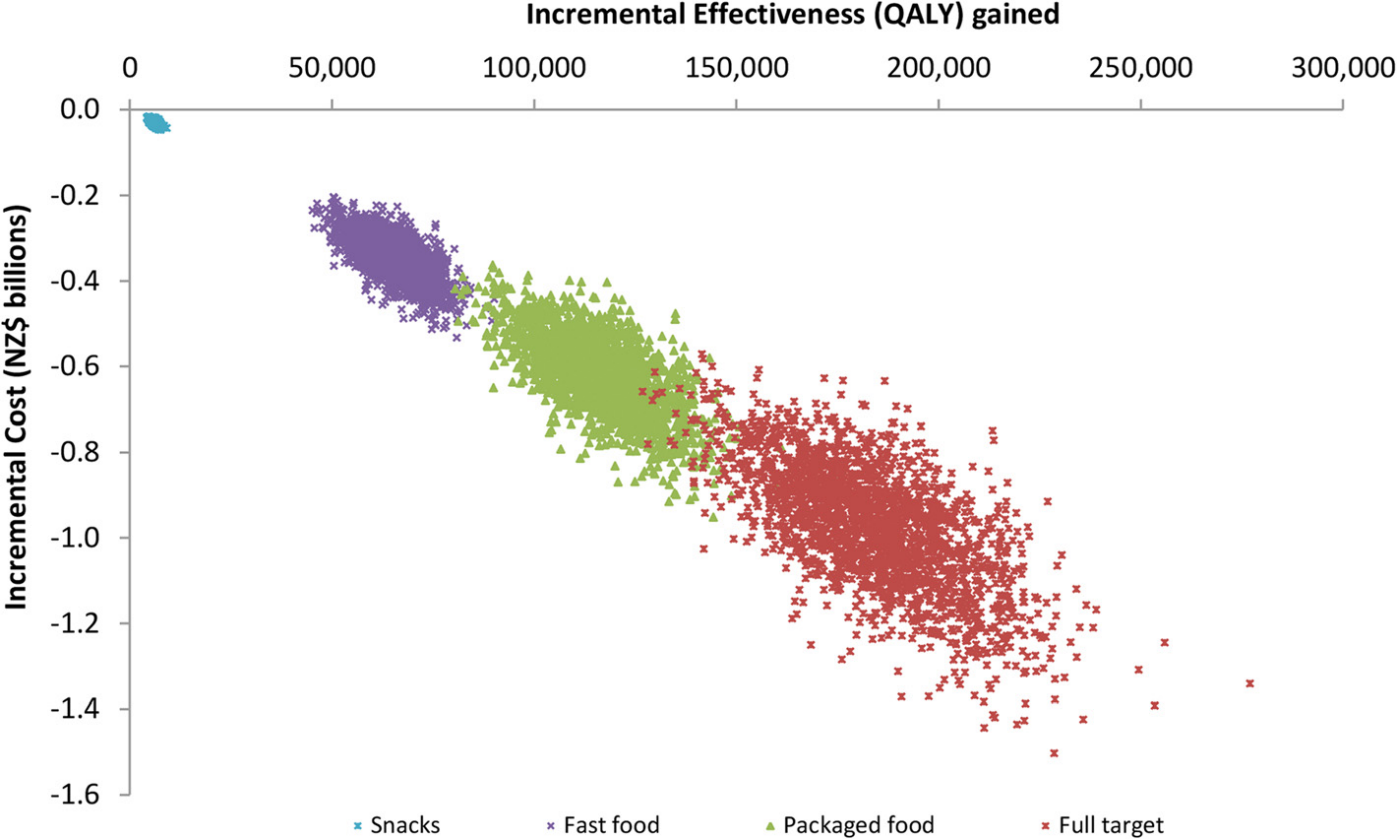
Cost-effectiveness plane for interventions that achieve selected mandatory sodium reduction targets for the New Zealand adult population (selected targets to show the full range of results)

The largest health gains were for achieving the “full target” with the mandatory approach (i.e., a 35 % relative reduction down to 5.5 g salt per day from reductions in packaged foods, and from fast food/restaurant foods, and from reduced discretionary use). That is, 235,000 QALYs gained over the lifetime of the modeled cohort (95 % uncertainty interval [UI]: 176,000 to 298,000; or a 0.7 % increase in lifetime QALYs for the 2011 cohort, i.e. 235,000/33.2 million) (Tables 3 and 4, Fig. 1). The results for the mandatory target were 6 % higher than for when this same target was achieved via a voluntary approach (at 222,000 QALYs gained). The health gain of various components of the full package in descending order and regardless of the mandatory or voluntary nature, was for achieving targets for: packaged foods; fast foods and restaurant meals; bread, sauces and processed meats combined; bread and bakery products; sauces; processed meats; bread; cheese; and snack food (the latter at 6100 QALYs for the mandatory approach).

**Table 3:** Population level results for the health gain and cost of the 10 sodium reduction interventions (95 % uncertainty intervals) ^a^

Intervention	Health gain (QALYs for remainder of the cohort’s life)	Health system cost (NZ$; millions) for remainder of the cohort’s life
“Do nothing” comparator ^b^	33.2 million (33.0 to 33.4 million)	162,000 (145,000 to 181,000)
Incremental to “Do nothing		
– Mandatory measures achieved		
1) Full target	235,000 (176,000 to 298,000)	-1260 (-1710 to 870)
2) Packaged foods target	122,000 (98,200 to 149,000)	-660 (-868 to 480)
3) Fast food & restaurant target	68,700 (55,200 to 83,600)	-370 (-487 to 270)
4) Bread target	8900 (7100 to 10,800)	-45.2 (-61 to 32)
5) Processed meats target	13,400 (10,800 to 16,200)	-70.0 (-94 to 50)
6) Sauces target	20,000 (16,100 to 24,300)	-106 (-141 to 77)
7) Package of Interventions 4 to 6	42,400 (34,200 to 51,500)	-228 (-302 to 167)
8) Snack food target	6100 (5000 to 7400)	-30.3 (-40 to 21)
9) All bread and bakery target	20,400 (16,600 to 24,800)	-108 (-141 to 78)
10) Cheese target	8800 (7100 to 10,600)	-44.6 (-59 to 32)
– Voluntary measures achieved		
1) Full target	222,000 (168,000 to 284,000)	-1170 (-1600 to 798)
2) Packaged foods target	115,000 (85,300 to 147,000)	-608 (-827 to 425)
3) Fast food & restaurant target	64,700 (48,000 to 82,200)	-338 (-461 to 236)
4) Bread target	8400 (6200 to 10,600)	-35.7 (-52 to 22)
5) Processed meats target	12,700 (9600 to 16,000)	-58.4 (-82 to 38)
6) Sauces target	18,900 (14,400 to 23,900)	-91.9 (-128 to 61)
7) Package of Interventions 4 to 6	40,100 (30,500 to 50,700)	-205 (-281 to 141)
8) Snack food target	5800 (4400 to 7300)	-22.0 (-34 to 12)
9) All bread and bakery target	19,400 (14,700 to 24,400)	-95.1 (-134 to 63)
10) Cheese target	8300 (6300 to 10,500)	-35.4 (-52 to 22)

^a^ Expected values for the NZ adult population aged 35+ years and alive in 2011 modeled out to death or age 100. Numbers are rounded to two or three meaningful digits

^b^ No intervention costs are included in this “do nothing comparator” (i.e., the costs of the currently existing programs of “dietary counselling by dietitians” and the “Endorsement Label Program” [4] are removed)

**Table 4:** Net health gain and costs incremental to “do nothing” by socio-demographic group for selected^a^ sodium reduction interventions (expressed per adult in 2011, over the remainder of their life with 3 % discounting)

	Full target (mandatory)		Packaged foods target (mandatory)		Fast foods & restaurant target (mandatory)		Snacks target (mandatory)	
Intervention/population group	Health gain (QALYs) per adult	Cost per adult (NZ$)	Health gain (QALYs) per adult	Cost per adult (NZ$)	Health gain (QALYs) per adult	Cost per adult (NZ$)	Health gain (QALYs) per adult	Cost per adult (NZ$)
Age < 65 years^b^	0.112	-$689	0.059	-$362	0.033	-$203	0.0029	-$16.9
Age 65+ years^b^	0.072	-$127	0.037	-$66	0.021	-$36.6	0.0019	-$2.2
Women	0.085	-$423	0.044	-$222	0.025	-$125	0.0022	-$9.9
Men	0.121	-$680	0.063	-$357	0.035	-$200	0.0032	-$16.8
Māori	0.130	-$461	0.068	-$242	0.038	-$136	0.0034	-$11.0
Non-Māori	0.099	-$555	0.051	-$291	0.029	-$163	0.0026	-$13.4

^a^ Interventions selected to show the three highest impact ones and also the lowest impact one (for the mandatory range of interventions)

^b^ This is the starting age-group, with the results for the rest of the lives in these modeled populations

Costs were strongly and linearly associated with QALYs, as the intervention costs were usually small meaning that avoided future health system costs were vastly more important, and reduced disease incidence co-generates QALY gains and reduced health system costs. All interventions were cost-saving (Table 3), with this being highest for the mandatory approach to the full target at $1260 million saved over the lifetime of the modeled cohort (compared to $1170 million for the full target via the voluntary approach). A cost-effectiveness plane in Fig. 1 shows results, with uncertainty, for a range of the mandatory interventions.

For all interventions, the health gains were greater for mandatory compared with voluntary approaches. The benefit was also greater per capita for men and for Māori e.g., 25 % more than non-Māori for the full target (see examples in Table 4). For all interventions in all socio-demographic groups, the interventions were also cost-saving.

The overall cost results were largely driven by averted disease treatment costs for CVD, followed by the increased health system costs from extra lives lived (as a result of the interventions) (Table D in Additional file 1).

### Scenario analyses

Scenario analyses showed that even with a higher 6 % discount rate, all interventions (and both mandatory and voluntary approaches) still produced net health system cost savings (Table E in Additional file 1). Net cost savings also occurred when we modeled an effect size that was half of the baseline values for the voluntary approach (to account for plausible non-compliance with the targets, Table F in Additional file 1).

### Uncertainty analyses

Tornado plots demonstrate how uncertainty in input parameters had an impact on the uncertainty in the model’s outputs (Figure A in Additional file 1 for the “packaged foods target” intervention). Uncertainty for costs was particularly driven by the uncertainty in the level of CHD disease cost (especially for men) and the effect size of the BP change for CHD risk.

For QALY gains, the most important drivers of modeled uncertainty were the extent of the sodium reduction (especially in men) and the effect size of the BP change for CHD risk and stroke risk.

## Discussion

### Main findings and interpretation

This study adds to the existing modeling-level evidence that interventions for reducing dietary sodium have the potential to generate large health gains and also large cost-savings for a health system. It suggests that there are large differences in the health and cost impact of the different specific targets, along with notable levels of uncertainty. Of the specific components of the full target intervention, it would seem the priority ones for maximizing health gain are the categories of “all packaged foods”, and then “fast foods and restaurant meals” (achieving 52 and 29 % of the health gain of the full target respectively). This pattern is similar to that found in a US modeling study [38].

Although these interventions based on reformulation are promising, other salt reduction interventions using the same model produce even larger health gain and greater cost savings: a sinking lid on the supply of salt and a salt tax [4]. Indeed, such salt reduction interventions may produce even greater health gains than regular increases in tobacco tax – at least for New Zealand [29]. However, reformulation interventions are particularly important to study because they may be more politically acceptable than other measures.

Mandatory approaches to achieving the targets were estimated to generate more health gain and be more cost-effective than voluntary ones. This pattern has been reported by others (see studies in Table 1 for Australia, England and New Zealand). Nevertheless, such differences may partly reflect various assumptions around intervention phase-in periods and intervention costs.

All interventions modeled were cost-saving and health inequality reducing as they generated greater QALY gains per adult for both the indigenous Māori population and men. This inequality reduction is similar to our previous work [4], and it reflects higher background CVD rates in Māori so that there is more potential for health gain from reducing blood pressure in Māori (there are minimal differences in sodium intakes between Māori and non-Māori). In contrast, no such pattern of inequality reduction by ethnic group has been reported for a salt-reduction intervention in the USA [38].

The collective results of this modeling work will probably have a reasonable applicability to other developed countries since high sodium intakes are common and a major risk to health internationally [1]. Nevertheless, the impact on health inequalities may vary between countries depending on population groups, background CVD risk, intake of processed foods, and genetic variation in salt sensitivity [39]. Countries can also differ in the levels of sodium in processed food [40]. Nonetheless, even with diverse food cultures in Europe, many of the countries are targeting similar types of food for salt reduction [14].

### Study strengths and limitations

This study built on previous New Zealand and Australian modeling work to explore a wide range of food category specific salt-reduction interventions. It appears to be only the second such modeling paper to consider such a large range of food categories (after Choi et al [38]) and also one of the few to use targets based on an evidence-based dietary salt reduction target model or specifically compare voluntary and mandatory approaches. The modeling work also benefited from relatively high quality cost and ethnicity data.

Nevertheless, there are limitations with all modeling studies, especially in comparison with well-designed experimental studies (although the latter are sometimes not possible for national-level policy interventions). As noted previously [4], there are also specific limitations with this sodium modeling work that should make policy-makers cautious in how they use any particular results. In summary these limitations include:

- Issues with model structure; and indeed our uncertainty estimates do not capture uncertainty arising from “model structure uncertainty”. For example, this model did not capture potential benefits of salt reduction on preventing stomach cancer [41] and renal disease [42]. Also, any potential benefits arising from the addition of extra potassium to processed food were not considered (as might be expected as per this systematic review [43] if food manufacturers replaced some sodium chloride with potassium chloride).
- Limitations around input parameters (e.g., particularly relating to the proportion of dietary sodium coming from fast food and restaurant meals and assumptions made in missing purchasing data for the dietary salt reduction target model). There were also limitations with current Health Tracker costs and some epidemiological data (e.g., prevalence of CVD [4]) and we also did not include uncertainty surrounding the phase-in periods for the interventions.
- Unknowns in how the public and industry might respond to achieving the targets were not captured. For example, the food industry might respond by adding flavor compensating ingredients such as more sugar, which could off-set some of the health benefits of sodium reductions (if sugar levels were also not regulated).

### Potential research and policy implications

Given this and other past work (*Introduction* and Table 1), the key research issue for most countries is probably to identify barriers (political and otherwise) to implementing various dietary salt reduction measures. If these barriers are considerable, then policy-makers could start with less ambitious reductions (but ideally still mandatory ones) via maximum sodium limits on various packaged foods. Or they could focus first on high sodium foods that can be considered to be non-essential (e.g., snack foods) or which have other health risks (e.g., the cancer risk from processed meats [44]). One way to potentially gain public support would be to combine the use of salt reduction targets with improvements in nutrient labeling and conducting mass media campaigns on the health hazard of high sodium diets. Finally, one way that stricter salt reduction targets might be more easily achieved is to encourage the food industry to use potassium salts as substitutes for sodium chloride (as used in Finland [45]).

Policy-makers who remain skeptical of the scientific basis for dietary sodium reduction and of the results of modeling studies have such choices as waiting for more evidence from experimental studies (e.g., a large trial underway in China [46]). Or they could focus on broader dietary interventions that may reduce the intake of dietary energy and free sugars as well as sodium (e.g., a Mexico-style “junk food” tax). Also if they wish to just focus on reducing the very high dietary intakes of sodium that virtually all researchers agree are hazardous (above 5000 mg sodium per day as per the Global Burden of Disease 2013 Study [1]), then they could target products that may particularly contribute to such high intakes (e.g., high sodium sauces).

## Conclusions

This work provides modeling-level evidence that achieving reduced sodium intakes via food category targets would generate large health gain and cost savings for the health sector. The categories of “all packaged foods”, then “fast foods and restaurant meals” appear to be the priority categories for achieving the largest health gains. These interventions may also be pro-equity by achieving larger per person benefits for men and indigenous populations.

## Additional file

Additional file 1: Additional Methods and Additional Results. (DOCX 96 kb)

## References

[1] Forouzanfar M, Alexander L, Anderson H, Bachman V, Biryukov S, Brauer M, Burnett R, Casey D, Coates M, GBD 2013 Risk Factors Collaborators (2015). Global, regional, and national comparative risk assessment of 79 behavioural, environmental and occupational, and metabolic risks or clusters of risks in 188 countries, 1990–2013: a systematic analysis for the Global Burden of Disease Study 2013. Lancet.

[2] Beaglehole R, Bonita R, Horton R, Adams C, Alleyne G, Asaria P, Baugh V, Bekedam H, Billo N, Casswell S (2011). Priority actions for the non-communicable disease crisis. Lancet.

[3] WHO: Guideline: Sodium intake for adults and children. Geneva, World Health Organization (WHO). http://www.who.int/nutrition/publications/guidelines/sodium_intake_printversion.pdf. (Accessed 22 Apr 2016.) 23658998

[4] Nghiem N, Blakely T, Cobiac LJ, Pearson AL, Wilson N (2015). Health and economic impacts of eight different dietary salt reduction interventions. PLoS One.

[5] Cook NR, Appel LJ, Whelton PK (2014). Lower levels of sodium intake and reduced cardiovascular risk. Circulation.

[6] Siervo M, Lara J, Chowdhury S, Ashor A, Oggioni C, Mathers JC. Effects of the Dietary Approach to Stop Hypertension (DASH) diet on cardiovascular risk factors: a systematic review and meta-analysis. Br J Nutr 2015;113(1):1-15. 10.1017/S0007114514003341 25430608

[7] Deckers IA, van den Brandt PA, van Engeland M, Soetekouw PM, Baldewijns MM, Goldbohm RA, Schouten LJ (2014). Long-term dietary sodium, potassium and fluid intake; exploring potential novel risk factors for renal cell cancer in the Netherlands Cohort Study on diet and cancer. Br J Cancer.

[8] Edwards DG, Farquhar WB (2015). Vascular effects of dietary salt. Curr Opin Nephrol Hypertens.

[9] Farquhar WB, Edwards DG, Jurkovitz CT, Weintraub WS (2015). Dietary Sodium and Health: More Than Just Blood Pressure. J Am Coll Cardiol.

[10] Poggio R, Gutierrez L, Matta MG, Elorriaga N, Irazola V, Rubinstein A (2015). Daily sodium consumption and CVD mortality in the general population: systematic review and meta-analysis of prospective studies. Public Health Nutr.

[11] Wang G, Labarthe D (2011). The cost-effectiveness of interventions designed to reduce sodium intake. J Hypertens.

[12] Wang G, Bowman BA (2013). Recent economic evaluations of interventions to prevent cardiovascular disease by reducing sodium intake. Curr Atheroscler Rep.

[13] World Health Organization: Creating an enabling environment for population-based salt reduction strategies: report of a joint technical meeting held by WHO and the Food Standards Agency, United Kingdom, July 2010. 2010. http://whqlibdoc.who.int/publications/2010/9789241500777_eng.pdf. (Accessed 22 Apr 2016)

[14] European Commission: Survey on members states implementation of the EU salt reduction framework: Directorate-General Health and Consumers, 2012. http://ec.europa.eu/health/nutrition_physical_activity/docs/salt_report1_en.pdf. (Accessed 22 Apr 2016).

[15] Webster J, Trieu K, Dunford E, Hawkes C (2014). Target salt 2025: a global overview of national programs to encourage the food industry to reduce salt in foods. Nutrients.

[16] Campbell N, Legowski B, Legetic B, Ferrante D, Nilson E, Campbell C, L’Abbe M (2014). Targets and timelines for reducing salt in processed food in the Americas. J Clin Hypertens.

[17] Charlton K, Webster J, Kowal P (2014). To legislate or not to legislate? A comparison of the UK and South African approaches to the development and implementation of salt reduction programs. Nutrients.

[18] He FJ, Brinsden HC, Macgregor GA (2014). Salt reduction in the United Kingdom: a successful experiment in public health. J Hum Hypertens.

[19] Brinsden HC, He FJ, Jenner KH, Macgregor GA. Surveys of the salt content in UK bread: progress made and further reductions possible. BMJ Open 2013;3(6):e002936. 10.1136/bmjopen-2013-002936 PMC3686219 23794567

[20] Eyles H, Webster J, Jebb S, Capelin C, Neal B, Ni Mhurchu C (2013). Impact of the UK voluntary sodium reduction targets on the sodium content of processed foods from 2006 to 2011: Analysis of household consumer panel data. Prev Med.

[21] Nghiem N, Wilson N, Blakely T. Technical Background to the Cardiovascular Disease Model used in the BODE^3^ Programme. Wellington: Department of Public Health, University of Otago. http://www.otago.ac.nz/wellington/otago070188.pdf; 2014. (Accessed 22 Apr 2016).

[22] Ministry of Health: Ways and Means: A report on methodology from the New Zealand Burden of Disease, Injury and Risk Study, 2006 - 2016. Wellington: Ministry of Health, 2013. http://www.health.govt.nz/publication/ways-and-means-report-methodology-new-zealand-burden-disease-injury-and-risk-study-2006–2016. (Accessed 22 Apr 2016).

[23] Woodward A, Blakely T (2014). The Healthy Country? A History of Life and Death in New Zealand.

[24] Blakely T, Foster R, Wilson N, BODE^3^ Team. Burden of Disease Epidemiology, Equity and Cost-Effectiveness (BODE3) Study Protocol. Version 2.1. Technical Report No.3. Wellington: Department of Public Health, University of Otago, Wellington, December 2012. http://www.otago.ac.nz/wellington/otago042986.pdf. (Accessed 22 Apr 2016).

[25] Baltussen R, Adam T, Tan-Torres Edejer T, Hutubessy R, Acharya A, et al. Methods for generalized cost-effectiveness analysis. In: Tan-Torres Edejer T, Baltussen R, Adam T, Hutubessy R, Acharya A, et al., editors. Making choices in health: WHO guide to cost-effectiveness analysis. Geneva: World Health Organization; 2003.

[26] Blakely T, Atkinson J, Kvizhinadze G, Nghiem N, McLeod H, Davies A, Wilson N (2015). Updated New Zealand health system cost estimates from health events by sex, age and proximity to death: further improvements in the age of ‘big data’. N Z Med J.

[27] Nghiem N, Wilson N, Blakely T. Validation Issues Relating to the Cardiovascular Disease Model Developed in the BODE^3^ Programme. Wellington: Department of Public Health, University of Otago; 2014. http://www.otago.ac.nz/wellington/otago070189.pdf. (Accessed 22 Apr 2016).

[28] Barendregt J, Oortmarssen GJ, Vos T, Murray CJL (2003). A generic model for the assessment of disease epidemiology: the computational basis of DisMod II. Popul Health Metr.

[29] Blakely T, Cobiac LJ, Cleghorn CL, Pearson AL, van der Deen FS, Kvizhinadze G, Nghiem N, McLeod M, Wilson N (2015). Health, health inequality, and cost impacts of annual increases in tobacco tax: Multistate life table modeling in New Zealand. PLoS Med.

[30] Salomon JA, Vos T, Hogan DR, Gagnon M, Naghavi M, Mokdad A, Begum N, Shah R, Karyana M, Kosen S (2012). Common values in assessing health outcomes from disease and injury: disability weights measurement study for the Global Burden of Disease Study 2010. Lancet.

[31] Shields E: Salt Reduction in New Zealand. The Development of a New Zealand Salt Reduction Model. Thesis for Master of Health Sciences in Nutrition and Dietetics, The University of Auckland, 2014. https://eshieldsdietitian.wordpress.com/. (Accessed 22 Apr 2016).

[32] Eyles H, Ni Mhurchu C (2014). Potential for electronic household food purchase data to enhance population nutrition monitoring. N Z Med J.

[33] University of Otago and Ministry of Health. A Focus on Nutrition: Key findings of the 2008/09 New Zealand Adult Nutrition Survey. Wellington: Ministry of Health; 2011. http://www.health.govt.nz/publication/focus-nutrition-key-findings-2008-09-nz-adult-nutrition-survey. (Accessed 22 Apr 2016).

[34] Centers for Disease Control and Prevention. Vital signs: food categories contributing the most to sodium consumption - United States, 2007–2008. MMWR. 2012;61(5):92–8. 22318472

[35] Department of Health: Salt Reduction 2017. United Kingdom, 2014. https://responsibilitydeal.dh.gov.uk/pledges/pledge/?pl=49. (Accessed 22 Apr 2016).

[36] Law MR, Frost CD, Wald NJ (1991). By how much does dietary salt reduction lower blood-pressure? 1. Analysis of observational data among populations. BMJ.

[37] Lewington S, Clarke R, Qizilbash N, Peto R, Collins R (2002). Age-specific relevance of usual blood pressure to vascular mortality: a meta-analysis of individual data for one million adults in 61 prospective studies. Lancet.

[38] Choi SE, Brandeau ML, Basu S (2016). Expansion of the National Salt Reduction Initiative: A mathematical model of benefits and risks of population-level sodium reduction. Med Decis Making.

[39] Doaei S, Gholamalizadeh M (2014). The association of genetic variations with sensitivity of blood pressure to dietary salt: A narrative literature review. ARYA Atheroscler.

[40] Dunford E, Webster J, Woodward M, Czernichow S, Yuan WL, Jenner K, Ni Mhurchu C, Jacobson M, Campbell N, Neal B (2012). The variability of reported salt levels in fast foods across six countries: opportunities for salt reduction. CMAJ.

[41] D’Elia L, Rossi G, Ippolito R, Cappuccio FP, Strazzullo P (2012). Habitual salt intake and risk of gastric cancer: a meta-analysis of prospective studies. Clin Nutr.

[42] Smyth A, O’Donnell MJ, Yusuf S, Clase CM, Teo KK, Canavan M, Reddan DN, Mann JF (2014). Sodium intake and renal outcomes: A systematic review. Am J Hypertens.

[43] Aburto NJ, Hanson S, Gutierrez H, Hooper L, Elliott P, Cappuccio FP (2013). Effect of increased potassium intake on cardiovascular risk factors and disease: systematic review and meta-analyses. BMJ.

[44] International Agency for Research on Cancer (World Health Organization): IARC Monographs evaluate consumption of red meat and processed meat. Lyon, France; International Agency for Research on Cancer, 2015.

[45] He FJ, Jenner KH, Macgregor GA (2010). WASH-world action on salt and health. Kidney Int.

[46] Li N, Yan LL, Niu W, Labarthe D, Feng X, Shi J, Zhang J, Zhang R, Zhang Y, Chu H, et al. A large-scale cluster randomized trial to determine the effects of community-based dietary sodium reduction--the China Rural Health Initiative Sodium Reduction Study. Am Heart J. 2013;166(5):815–22. 10.1016/j.ahj.2013.07.009 PMC3919684 24176436

[47] Cobiac LJ, Vos T, Veerman JL. Cost-effectiveness of interventions to reduce dietary salt intake. Heart. 2010;96(23):1920-1925. 10.1136/hrt.2010.199240 21041840

[48] Smith-Spangler CM, Juusola JL, Enns EA, Owens DK, Garber AM (2010). Population strategies to decrease sodium intake and the burden of cardiovascular disease: a cost-effectiveness analysis. Ann Int Med.

[49] Rubinstein A, Colantonio L, Bardach A, Caporale J, Marti SG, Kopitowski K (2010). Estimation of the burden of cardiovascular disease attributable to modifiable risk factors and cost-effectiveness analysis of preventative interventions to reduce this burden in Argentina. BMC Public Health..

[50] Rubinstein A, Garcia Marti S, Souto A, Ferrante D, Augustovski F. Generalized cost-effectiveness analysis of a package of interventions to reduce cardiovascular disease in Buenos Aires, Argentina. Cost Eff Resour Alloc. 2009;7:10 10.1186/1478-7547-7-10 PMC2684068 19419570

[51] Bertram MY, Steyn K, Wentzel-Viljoen E, Tollman S, Hofman KJ (2012). Reducing the sodium content of high-salt foods: effect on cardiovascular disease in South Africa. S Afr Med J.

[52] Cobiac LJ, Magnus A, Lim S, Barendregt JJ, Carter R, Vos T (2012). Which interventions offer best value for money in primary prevention of cardiovascular disease?. PLoS One.

[53] Konfino J, Mekonnen TA, Coxson PG, Ferrante D, Bibbins-Domingo K (2013). Projected impact of a sodium consumption reduction initiative in Argentina: an analysis from the CVD policy model--Argentina. PLoS One.

[54] Hendriksen MA, Hoogenveen RT, Hoekstra J, Geleijnse JM, Boshuizen HC, van Raaij JM (2014). Potential effect of salt reduction in processed foods on health. Am J Clin Nutr.

[55] Wilson N, Nghiem N, Eyles H, Ni Mhurchu C, Cobiac LJ, Pearson AL (2014). Possible impact of the Tick Programme in New Zealand on selected nutrient intakes: Tentative estimates and methodological complexities. N Z Med J.

[56] Gillespie DO, Allen K, Guzman-Castillo M, Bandosz P, Moreira P, McGill R (2015). The Health Equity and Effectiveness of Policy Options to Reduce Dietary Salt Intake in England: Policy Forecast. PLoS One.

[57] Jaenke R, Barzi F, McMahon E, Webster J, Brimblecombe J. Consumer Acceptance of Reformulated Food Products: A Systematic Review and Meta-analysis of Salt-reduced Foods. Crit Rev Food Sci Nutr. 2016:0. 10.1080/10408398.2015.1118009 26745848

