# Supplementary material for: Modeling health gains and cost savings for ten dietary salt reduction targets
Source: Nutr J. 2016 Apr 26;15:44. doi: 10.1186/s12937-016-0161-1 (PMC4847342; doi:10.1186/s12937-016-0161-1)
Supplement: Additional file 1: — Additional Methods and Additional Results. (DOCX 96 kb) [file 12937_2016_161_MOESM1_ESM.docx]

## Additional File

## Additional Methods

Table A. Input parameters to the modeling: selected baseline and epidemiological parameters

| **Variable** | **Sources and key details** | **Key values and uncertainty** |
| --- | --- | --- |
| ***Baseline variables*** | |  |
| Sodium intake | Source: Based on a representative sample of 300 NZ adults which indicated a mean sodium excretion of 3374 mg/d [[1](#_ENREF_1)]. This was a more recent study than that used in our previous model [[2](#_ENREF_2)] and had the advantage of being based on gold standard 24-hour urine samples as opposed to the spot urine samples in the national health survey. To determine the foods supplying sodium we used a combination of market research company data on food purchases (electronically scanned by a consumer panel) and “Nutritrack” data for nutritional composition data on packaged foods available in NZ supermarkets and food in fast food restaurants [[3](#_ENREF_3)]. Further work was done to develop a New Zealand dietary salt reduction “target model” which more fully determined the sodium contribution from different food sources (e.g., adjusting for discretionary salt use and for various fresh foods) [[4](#_ENREF_4)]. This work also included food category specific target components (building partly on the development of the UK salt reduction model which had benefited from extensive consultation with the food industry [[5](#_ENREF_5)]).  We assumed no future trend in sodium intake, given no notable trend since the 1980s for NZ [[6](#_ENREF_6)]. | 3374 mg/d  Nil uncertainty; rather uncertainty around the intervention associated reduction was considered – see below. |
| Incidence, prevalence and case-fatality data for CHD and stroke | Calculated using linked Health Tracker data, with coherency checks using DisMod II and smoothing with regression as required. Future annual percentage change (APC) in incidence and CFR were both set at -2.0% each as per the NZBDS. | See online reports for details: [[7](#_ENREF_7), [8](#_ENREF_8)]. |
| Morbidity (disability weights [DW]) | From GBD2010 [[9](#_ENREF_9)], with modification to NZ [[10](#_ENREF_10)] and slight variation by age and ethnicity (see an online report [[7](#_ENREF_7)] for details). | CHD = 0.081  Stroke = 0.226  Uncertainty: e.g., for non-Māori males, 95%CI: 0.05 – 0.11 for CHD and 0.11 – 0.23 for stroke. (For more details uncertainty see Nghiem et al [[7](#_ENREF_7)]). |
| Baseline health system costs for CHD and stroke states, and non-diseased states | Calculated from Health Tracker data by sex and age in 2011 for people: (a) without either CHD or stroke; (b) with CHD only, and *excess* to (a); (c) with stroke only, and *excess* to (a). (See an online report [[7](#_ENREF_7)] for details). | Examples for 60 year old females (gamma distribution with SD=10% of mean): (a) NZ$2,381; (b) NZ$16,258 for the first year, NZ$5,395 for second and subsequent years; (c) NZ$20,553 and NZ$5,991 for stroke. |
| ***Epidemiological associations*** | |  |
| Change in systolic blood pressure (sBP) (in mm Hg) for each 100 mmol/d change in sodium intake | Derived from the regressions models developed by Law et al [[11](#_ENREF_11)]. The small differences in BP by ethnic group did not justify separate modeling by ethnicity (higher in Māori by 3 mm Hg for systolic BP and 4 mm Hg for diastolic BP in both sexes compared to non-Māori [[12](#_ENREF_12)]). Also of note is that no trend in BP into the future was considered given the unclear picture in NZ (of a downward trend in population BP levels from 1982 to 2002 and then an upward trend from then 2008/09) [[12](#_ENREF_12)]. | For men and women:  Age-group; sBP (mm Hg) change  30-39: 5.5  40-49: 6.6  50-59: 9.2  60-69: 10.3 |
| Relationship between blood pressure and CVD risks | We used the results of a meta-analysis of 61 prospective studies by Lewington et al [[13](#_ENREF_13)]. These results were considered to be more generalisable to the general population than those from a meta-analysis by Law et al 2009 of 147 RCTs of blood pressure-lowering drugs [[14](#_ENREF_14)]. | The hazard ratio for a 20 mm Hg reduction in systolic BP ranged from 0.49 to 0.67 for CHD and from 0.38 to 0.67 for stroke (depending on age). For uncertainty: SD = +/- 10% of the point estimate for each age group. |

Table B. Input parameters relating to the effects of the 10 food target-related interventions

| **Intervention** | **Extra details** | **Key values and uncertainty (average adult)^a^** |
| --- | --- | --- |
| 1) Full target achieved (i.e., 35% relative reduction to 5.5 g/d), from: packaged food categories plus fast food and reduced discretionary use | **Targets:** The combination of the Intervention 2 target below (36% reduction in sodium), plus the Intervention 3 target (40% reduction), plus discretionary use reduction (40% reduction).  **Phasing in:** The effect would be phased in linearly over a 5 year period (for the voluntary approach) and over 3 years for the mandatory approach. | Given no change in fresh foods, this is equivalent to an overall reduction of 35% of sodium intake (or 1185 mg [51.5 mmol] sodium per day for an average adult). For uncertainty we used SD of +/- 15% of the point estimate (normal distribution). |
| 2) Packaged foods target achieved | **Targets:** The specific targets for all packaged foods – including those packaged foods in this table and others in the full model [[4](#_ENREF_4)] (a 36% reduction in sodium in these foods overall).  **Phasing in:** As per Intervention 1. | Reduction of 628 mg (27.3 mmol) sodium per day for an average adult. For uncertainty: SD of +/- 10% of the point estimate (normal distribution) if mandatory and SD of +/-15% if voluntary. |
| 3) Fast food and restaurant target achieved | **Targets:** An overall reduction of 40% in sodium in these foods.  **Phasing in:** As per Intervention 1. | Reduction of 355 mg (15.4 mmol) sodium per day for an average adult. For uncertainty: as per Intervention 2. |
| 4) Bread target achieved | **Targets:** Targets ranged from a 12% reduction in wholemeal bread to a 37% reduction in “other bread”. The most common target was to reduce to 350mg sodium per 100g of bread.  **Phasing in:** The effect would be phased in linearly over a 5 year period (for the voluntary approach) and over 3 years for the mandatory approach. | Reduction of 43.6 mg (2.0 mmol) sodium per day for an average adult. For uncertainty: as per Intervention 2. |
| 5) Processed meats target achieved | **Targets:** The range of specific targets was from a 35% reduction in “cured meats” to a 55% reduction in “other meat products” (covering all categories except for “raw” and “frozen” meat).  **Phasing in:** As per Intervention 4. | Reduction of 69.9 mg (3.0 mmol) sodium per day for an average adult. For uncertainty: as per Intervention 2. |
| 6) Sauces target achieved | **Targets:** The range of specific targets was from a 30% reduction in marinades to a 63% reduction in “powdered mixes for meal-based sauces”.  **Phasing in:** As per Intervention 4. | Reduction of 104 mg (4.5 mmol) sodium per day for an average adult. For uncertainty: as per Intervention 2. |
| 7) Package of Interventions 4 to 6 | The combined (fully additive) effect of achieving the targets for bread, processed meats and sauces collectively (the top three groupings for sources of dietary sodium). | Reduction of 217.5 mg (9.50 mmol) sodium per day for an average adult. For uncertainty: as per Intervention 2. |
| 8) Snack food target achieved | **Targets:** The range of specific targets was from a 34% reduction in “extruded snacks” to a 48% reduction in “potato chips”.  **Phasing in:** As per Intervention 4. | Reduction of 32.2 mg (1.4 mmol) sodium per day for an average adult. For uncertainty: as per Intervention 2. |
| 9) All bread and bakery target achieved | **Targets:** As per Intervention 4 but with all other bakery products added (54% reduction in “sweet biscuits” and 63% reduction in “cakes, muffins and pastries”).  **Phasing in:** As per Intervention 4. | Reduction of 107 mg (4.6 mmol) sodium per day for an average adult. For uncertainty: as per Intervention 2. |
| 10) Cheese target achieved | **Targets:** The range of specific targets was from a 27% reduction in “hard block cheese” to a 42% reduction in “soft/fresh cheese”.  **Phasing in:** As per Intervention 4. | Reduction of 45.8 mg (2.0 mmol) sodium per day for an average adult. For uncertainty: as per Intervention 2. |

^a^ Values given for the average adult. In the modeling we adjusted these values for men and women by ratios of 4013/3544 mg and 3115/3544 mg respectively, given the variation in sodium intakes (in mg) according to national nutrition survey data [[15](#_ENREF_15)].

Table C. Input parameters relating to the interventions costs

| **Intervention** | **Intervention costs – voluntary route** | **Intervention costs – mandated route** |
| --- | --- | --- |
| 1) Full target achieved (compatible with 35% relative reduction to 5.5 g/d if discretionary intake reduction also achieved) | The equivalent of running the Heart Foundation’s “Tick Program” for 10 years. That is $NZ 621,000 per year for 10 years. This is the annual cost reported for the calendar year 2011 (see an online report [[16](#_ENREF_16)]). For uncertainty we used a gamma distribution with SD of +/-10% of the point-estimate. | The cost was just the cost of a new law for NZ which was based on the average cost of new act [[17](#_ENREF_17)] (with NZ$ dollar values reported separately [[18](#_ENREF_18)]). That is, the cost of a new law was estimated NZ$ 3,680,000 (in 2011 dollars). For uncertainty we used a gamma distribution with SD of +/-25% of the point-estimate. While the cost of the law is relevant for changes in packaged foods and the fast food sector, for the additional reduction in “discretionary” salt (e.g., at the table) we assumed that there was no specific extra cost for promoting this as it was assumed to be achieved via the publicity associated with the new laws and on-going publicity from existing health agencies (e.g., the Ministry of Health).  Reformulation costs and costs associated with package labelling changes was considered out-of-scope given our health system perspective. Such an approach has been taken previously with new laws in NZ relating to food labelling, alcohol labelling and tobacco labelling in that manufacturers are not compensated for the costs imposed by the new law. For example, the NZ law requiring pictorial health warnings on tobacco packaging did not compensate industry for printing costs or lost sales. Furthermore, we assumed no additional costs from the existing routine evaluation efforts by the NZ Government (nutrition surveys and food surveys) and negligible enforcement and legal costs associated with non-compliance (owing to the low levels of corruption in the NZ setting and the high compliance with laws e.g., the law banning smoking in bars and restaurants [[19](#_ENREF_19)]). |
| 2) Packaged foods target achieved | As above. | As per Intervention 1, i.e., a one-off law cost. |
| 3) Fast food and restaurant target achieved | As above. | As per Intervention 1, i.e., a one-off law cost. |
| 4) Bread target achieved | As above except for half the cost ($310,000) and with this spread over five years (given the reduced scale of the industries involved). | As per Intervention 1, i.e., a one-off law cost. |
| 5) Processed meats target achieved | As per Intervention 4 (i.e., for 5 years). | As per Intervention 1, i.e., a one-off law cost. |
| 6) Sauces target achieved | As per Intervention 4 (i.e., for 5 years). | As per Intervention 1, i.e., a one-off law cost. |
| 7) Package of Interventions 4 to 6 | As above for interventions 4 to 6. | As per Intervention 1, i.e., a one-off law cost. |
| 8) Snack food target achieved | As per Intervention 4 (i.e., for 5 years). | As per Intervention 1, i.e., a one-off law cost. |
| 9) All bread and bakery target achieved | As per Intervention 4 (i.e., for 5 years). | As per Intervention 1, i.e., a one-off law cost. |
| 10) Cheese target achieved | As per Intervention 4 (i.e., for 5 years). | As per Intervention 1, i.e., a one-off law cost. |

## Additional Results

Table D. Types of costs (NZ$) for selected sodium reduction interventions (expressed per adult in 2011)

| **Intervention** | **Direct intervention cost** | **CVD health system costs** | **Non-CVD health system costs** | **Net cost** |
| --- | --- | --- | --- | --- |
| ***Baseline*** |  |  |  |  |
| “Do nothing” comparator | – | 16,000 | 54,500 | 70,500 |
| Full target (mandatory) | 1.43 | 14,900 | 55,000 | 69,900 |
| Packaged foods target (mandatory) | 1.43 | 15,400 | 54,800 | 70,200 |
| Fast food & restaurant target (mandatory) | 1.43 | 15,700 | 54,700 | 70,300 |
| Snack food target (mandatory) | 1.43 | 15,900 | 54,500 | 70,500 |
| ***Incremental to “do nothing” costs*** |  |  |  |  |
| Full target (mandatory) | 1.43 | -1,070 | 525 | -546 |
| Packaged foods target (mandatory) | 1.43 | -561 | 273 | -287 |
| Fast food & restaurant target (mandatory) | 1.43 | -315 | 153 | -161 |
| Snack food target (mandatory) | 1.43 | -28.4 | 13.7 | -13.2 |

Table E: Scenario analysis involving differing discount rates for the target sodium reduction interventions (health gain and costs)

| **Interventions** | **Discount rate** | **Total health gain (QALYs)** | **Costs (NZ$ million)** | **Health gain per adult (QALYs)** | **Incremental cost per adult (NZ$)** |
| --- | --- | --- | --- | --- | --- |
| ***Mandatory approach*** |  |  |  |  |  |
| “Do nothing” comparator | 0% | 51,600,000 | $287,000 | N/A | N/A |
| 1) Full target | 0% | 553,000 | -$1,310 | 0.240 | -$569 |
| 2) Packaged foods target | 0% | 288,000 | -$689 | 0.125 | -$299 |
| 3) Fast food & restaurant target | 0% | 161,000 | -$387 | 0.070 | -$168 |
| 4) Bread target | 0% | 20,800 | -$47 | 0.009 | -$21 |
| 5) Processed meats target | 0% | 31,500 | -$73 | 0.014 | -$32 |
| 6) Sauces target | 0% | 47,000 | -$111 | 0.020 | -$48 |
| 7) Package of Interventions 4 to 6 | 0% | 99,700 | -$239 | 0.043 | -$104 |
| 8) Snack food target | 0% | 14,500 | -$32 | 0.006 | -$14 |
| 9) All bread and bakery target | 0% | 48,100 | -$114 | 0.021 | -$49 |
| 10) Cheese target | 0% | 20,600 | -$47 | 0.009 | -$20 |
| “Do nothing” comparator | 3% | 33,200,000 | $162,000 | N/A | N/A |
| 1) Full target | 3% | 234,000 | -$1,260 | 0.102 | -$546 |
| 2) Packaged foods target | 3% | 122,000 | -$659 | 0.053 | -$286 |
| 3) Fast food & restaurant target | 3% | 68,600 | -$370 | 0.030 | -$161 |
| 4) Bread target | 3% | 8,870 | -$45 | 0.004 | -$20 |
| 5) Processed meats target | 3% | 13,400 | -$70 | 0.006 | -$30 |
| 6) Sauces target | 3% | 20,000 | -$106 | 0.009 | -$46 |
| 7) Package of Interventions 4 to 6 | 3% | 42,500 | -$228 | 0.018 | -$99 |
| 8) Snack food target | 3% | 6,160 | -$30 | 0.003 | -$13 |
| 9) All bread and bakery target | 3% | 20,500 | -$109 | 0.009 | -$47 |
| 10) Cheese target | 3% | 8,780 | -$45 | 0.004 | -$19 |
| “Do nothing” comparator | 6% | 24,000,000 | $106,000 | N/A | N/A |
| 1) Full target | 6% | 116,000 | -$973 | 0.050 | -$423 |
| 2) Packaged foods target | 6% | 60,500 | -$510 | 0.026 | -$222 |
| 3) Fast food & restaurant target | 6% | 34,000 | -$286 | 0.015 | -$124 |
| 4) Bread target | 6% | 4,400 | -$34 | 0.002 | -$15 |
| 5) Processed meats target | 6% | 6,650 | -$53 | 0.003 | -$23 |
| 6) Sauces target | 6% | 9,940 | -$81 | 0.004 | -$35 |
| 7) Package of Interventions 4 to 6 | 6% | 21,000 | -$176 | 0.009 | -$76 |
| 8) Snack food target | 6% | 3,060 | -$23 | 0.001 | -$10 |
| 9) All bread and bakery target | 6% | 10,200 | -$83 | 0.004 | -$36 |
| 10) Cheese target | 6% | 4,360 | -$34 | 0.002 | -$15 |
| **Voluntary approach** |  |  |  |  |  |
| “Do nothing” comparator | 0% | 51,600,000 | $287,000 | N/A | N/A |
| 1) Full target | 0% | 533,000 | -$1,230 | 0.232 | -$534 |
| 2) Packaged foods target | 0% | 278,000 | -$641 | 0.121 | -$279 |
| 3) Fast food & restaurant target | 0% | 156,000 | -$355 | 0.068 | -$154 |
| 4) Bread target | 0% | 20,100 | -$34 | 0.009 | -$15 |
| 5) Processed meats target | 0% | 30,400 | -$58 | 0.013 | -$25 |
| 6) Sauces target | 0% | 45,400 | -$94 | 0.020 | -$41 |
| 7) Package of Interventions 4 to 6 | 0% | 96,200 | -$215 | 0.042 | -$93 |
| 8) Snack food target | 0% | 14,000 | -$19 | 0.006 | -$8 |
| 9) All bread and bakery target | 0% | 46,500 | -$97 | 0.020 | -$42 |
| 10) Cheese target | 0% | 19,900 | -$33 | 0.009 | -$14 |
| “Do nothing” comparator | 3% | 33,200,000 | $162,000 | N/A | N/A |
| 1) Full target | 3% | 222,000 | -$1,170 | 0.096 | -$508 |
| 2) Packaged foods target | 3% | 116,000 | -$610 | 0.050 | -$265 |
| 3) Fast food & restaurant target | 3% | 65,000 | -$340 | 0.028 | -$148 |
| 4) Bread target | 3% | 8,400 | -$36 | 0.004 | -$16 |
| 5) Processed meats target | 3% | 12,700 | -$59 | 0.006 | -$26 |
| 6) Sauces target | 3% | 19,000 | -$93 | 0.008 | -$40 |
| 7) Package of Interventions 4 to 6 | 3% | 40,200 | -$207 | 0.018 | -$90 |
| 8) Snack food target | 3% | 5,840 | -$22 | 0.003 | -$10 |
| 9) All bread and bakery target | 3% | 19,400 | -$95 | 0.008 | -$41 |
| 10) Cheese target | 3% | 8,320 | -$35 | 0.004 | -$15 |
| “Do nothing” comparator | 6% | 24,000,000 | $106,000 | N/A | N/A |
| 1) Full target | 6% | 107,000 | -$889 | 0.047 | -$386 |
| 2) Packaged foods target | 6% | 56,200 | -$465 | 0.024 | -$202 |
| 3) Fast food & restaurant target | 6% | 31,600 | -$259 | 0.014 | -$112 |
| 4) Bread target | 6% | 4,080 | -$28 | 0.002 | -$12 |
| 5) Processed meats target | 6% | 6,170 | -$45 | 0.003 | -$20 |
| 6) Sauces target | 6% | 9,220 | -$71 | 0.004 | -$31 |
| 7) Package of Interventions 4 to 6 | 6% | 19,500 | -$158 | 0.009 | -$69 |
| 8) Snack food target | 6% | 2,840 | -$17 | 0.001 | -$7 |
| 9) All bread and bakery target | 6% | 9,440 | -$73 | 0.004 | -$32 |
| 10) Cheese target | 6% | 4,040 | -$27 | 0.002 | -$12 |

Table F: Scenario analysis involving reduced effectiveness for the voluntary approach to achieving the target sodium reduction interventions (i.e., half of the effectiveness to account for plausible non-compliance with the targets, otherwise same as baseline).

| **Interventions** | **Total health gain (QALYs)** | **Costs (NZ$ millions)** | **Health gain per adult (QALYs)** | **Incremental cost per adult (NZ$)** |
| --- | --- | --- | --- | --- |
| “Do nothing” comparator | 33,200,000 | $162,000 | N/A | N/A |
| 1) Full target | 109,000 | -$575 | 0.048 | -$250 |
| 2) Packaged foods target | 57,500 | -$299 | 0.025 | -$130 |
| 3) Fast food & restaurant target | 32,400 | -$165 | 0.014 | -$71.5 |
| 4) Bread target | 4,200 | -$13 | 0.0018 | -$5.7 |
| 5) Processed meats target | 6,340 | -$25 | 0.0028 | -$10.8 |
| 6) Sauces target | 9,480 | -$42 | 0.0041 | -$18.1 |
| 7) Package of Interventions 4 to 6 | 20,100 | -$99 | 0.0087 | -$42.8 |
| 8) Snack food target | 2,920 | -$6 | 0.0013 | -$2.7 |
| 9) All bread and bakery target | 9,700 | -$43 | 0.0042 | -$18.6 |
| 10) Cheese target | 4,160 | -$13 | 0.0018 | -$5.6 |

#

# Uncertainty analysis – tornado plots

Figure A: Tornado plots for univariate sensitivity analyses using the 2.5^th^ and 97.5^th^ percentile of input parameters on incremental cost and QALYs gained per individual NZ adult (“packaged foods target” using a mandatory approach)

1. Incremental cost, compared to “do nothing”

1. Incremental QALYs, compared to “do nothing”

**References**

1. Ministry for Primary Industries: The impact of mandatory fortification of bread with iodine. MPI Technical Paper No: 2013/025. Wellington: Ministry for Primary Industries, New Zealand Government. <http://www.foodsafety.govt.nz/elibrary/industry/mandatory-fortification-bread-iodine.pdf;> 2013.

2. Nghiem N, Blakely T, Cobiac LJ, Pearson AL, Wilson N: Health and economic impacts of eight different dietary salt reduction interventions. PLoS One 2015, 10(4):e0123915.

3. Eyles H, Ni Mhurchu C: Potential for electronic household food purchase data to enhance population nutrition monitoring. N Z Med J 2014, 127(1403):68-71.

4. Shields E: Salt Reduction in New Zealand. The Development of a New Zealand Salt Reduction Model. Thesis for Master of Health Sciences in Nutrition and Dietetics, The University of Auckland. https://eshieldsdietitian.wordpress.com/. 2014.

5. Department of Health: Salt Reduction 2017. United Kingdom, 2014. https://responsibilitydeal.dh.gov.uk/pledges/pledge/?pl=49.

6. McLean RM, Mann JI, Hoek J: World Salt Awareness Week: more action needed in New Zealand. N Z Med J 2011, 124(1332):68-76.

7. Nghiem N, Wilson N, Blakely T: Technical Background to the Cardiovascular Disease Model used in the BODE³ Programme. Wellington: Department of Public Health, University of Otago. <http://www.otago.ac.nz/wellington/otago070188.pdf;> 2014.

8. Nghiem N, Wilson N, Blakely T: Validation Issues Relating to the Cardiovascular Disease Model Developed in the BODE³ Programme. Wellington: Department of Public Health, University of Otago. <http://www.otago.ac.nz/wellington/otago070189.pdf;> 2014.

9. Salomon JA, Vos T, Hogan DR, Gagnon M, Naghavi M, Mokdad A, Begum N, Shah R, Karyana M, Kosen S et al: Common values in assessing health outcomes from disease and injury: disability weights measurement study for the Global Burden of Disease Study 2010. Lancet 2012, 380(9859):2129-2143.

10. Ministry of Health: Ways and Means: A report on methodology from the New Zealand Burden of Disease, Injury and Risk Study, 2006 - 2016. Wellington: Ministry of Health. <http://www.health.govt.nz/publication/ways-and-means-report-methodology-new-zealand-burden-disease-injury-and-risk-study-2006-2016;> 2013.

11. Law MR, Frost CD, Wald NJ: By how much does dietary salt reduction lower blood-pressure? 1. Analysis of observational data among populations. BMJ 1991, 302(6780):811-815.

12. McLean RM, Williams S, Mann JI, Miller JC, Parnell WR: Blood pressure and hypertension in New Zealand: results from the 2008/09 Adult Nutrition Survey. N Z Med J 2013, 126(1372):1-14.

13. Lewington S, Clarke R, Qizilbash N, Peto R, Collins R: Age-specific relevance of usual blood pressure to vascular mortality: a meta-analysis of individual data for one million adults in 61 prospective studies. Lancet 2002, 360(9349):1903-1913.

14. Law MR, Morris JK, Wald NJ: Use of blood pressure lowering drugs in the prevention of cardiovascular disease: meta-analysis of 147 randomised trials in the context of expectations from prospective epidemiological studies. BMJ 2009, 338(191):b1665.

15. McLean R, Williams S, Mann J, Parnell W: How much salt are we eating? Estimates of New Zealand population sodium from the 2008/2009 Adult Nutrition Survey [Presentation on 2 December 2011]. Joint Annual Scientific Meeting of the Australian and New Zealand Nutrition Societies. Queenstown, New Zealand (29 November - 2 December); 2011.

16. Wilson N, Nghiem N: Background Report for BODE3 Modelling on Estimating the Impact of the Tick Programme in New Zealand (a Heart Health Food Endorsement Programme). Wellington: University of Otago, Wellington. <http://www.otago.ac.nz/wellington/otago071961.pdf;> 2014.

17. Wilson N, Nghiem N, Foster R, Cobiac L, Blakely T: Estimating the cost of new public health legislation. Bull World Health Organ 2012, 90:532-539.

18. BODE3 Programme: Results for the cost of making a new law, all in $NZ. <http://www.otago.ac.nz/wellington/otago033080.pdf>. 2012.

19. Wilson N, Edwards R, Parry R: A persisting secondhand smoke hazard in urban public places: results from fine particulate (PM2.5) air sampling. N Z Med J 2011, 124(1330):34-47.
